# *gef* Gene Expression in MCF-7 Breast Cancer Cells is Associated with a Better Prognosis and Induction of Apoptosis by p53-Mediated Signaling Pathway

**DOI:** 10.3390/ijms12117445

**Published:** 2011-10-31

**Authors:** Houria Boulaiz, Pablo J. Álvarez, Jose Prados, Juan Marchal, Consolación Melguizo, Esmeralda Carrillo, Macarena Peran, Fernando Rodríguez, Alberto Ramírez, Raúl Ortíz, Antonia Aránega

**Affiliations:** 1 Basic Cardiovascular Research Section, Department of Anatomy and Human Embriology, School of Medicine, University of Granada, Granada E-18012, Spain; E-Mails: pablo.alvarez.ext@juntadeandalucia.es (P.J.A.); jcpradois@ugr.es (J.P.); marchal@ugr.es (J.M.); cmelguizo@ugr.es (C.M.); esmeralda@ugr.es (E.C.); fernr@ugr.es (F.R.); 2 Biopathology and Medicine Regenerative Institute (IBIMER), Granada 18100, Spain; E-Mails: alrari8@gmail.com (A.R.); Ortizr@ujaen.es (R.O.); 3 Department of Health Sciences, University of Jaén, E-23071 Jaén, Spain; E-Mail: peran@ujaen.es

**Keywords:** breast cancer, *gef* gene, tumor markers, loss of tumor malignancy

## Abstract

Breast cancer research has developed rapidly in the past few decades, leading to longer survival times for patients and opening up the possibility of developing curative treatments for advanced breast cancer. Our increasing knowledge of the biological pathways associated with the progression and development of breast cancer, alongside the failure of conventional treatments, has prompted us to explore gene therapy as an alternative therapeutic strategy. We previously reported that *gef* gene from *E. coli* has shown considerable cytotoxic effects in breast cancer cells. However, its action mechanism has not been elucidated. Indirect immunofluorescence technique using flow cytometry and immunocytochemical analysis were used to detect breast cancer markers: estrogen (ER) and progesterone (PR) hormonal receptors, human epidermal growth factor receptor-2 proto-oncogene (c-erbB-2), ki-67 antigen and p53 protein. *gef* gene induces an increase in ER and PR expressions and a decrease in ki-67 and c-erbB-2 gene expressions, indicating a better prognosis and response to treatment and a longer disease-free interval and survival. It also increased p53 expression, suggesting that gef-induced apoptosis is regulated by a p53-mediated signaling pathway. These findings support the hypothesis that the *gef* gene offers a new approach to gene therapy in breast cancer.

## 1. Introduction

Breast cancer is the most common malignant tumor and the highest cause of death by neoplasia among women [1]. It is correlated with multiple histopathological forms, growth rates, variations in metastatic capacity and responses to hormonal therapy. All of these variations are a reflection of numerous genomic modifications that take place during tumor transformation [2,3]. Uncontrolled cell proliferation and/or apoptosis can lead to breast cancer as a consequence of the accumulation of genetic lesions, giving rise to alterations that activate protooncogenes and inactivate tumor suppressor genes [4,5]. The most significant conventional prognostic factors for breast cancer are tumor size, status of axillary lymph nodes, degree of differentiation and classification of the neoplasia, and a panel of specific tumor markers [6,7]. In this context, the most widely used breast cancer markers are estrogen (ER) and progesterone (PR) hormonal receptors, human epidermal growth factor receptor-2 proto-oncogene (Her-2/neu or c-erbB-2), ki-67 antigen and p53 protein [1,8–10]. The prognostic markers ER and PR are used to select treatments with greater clinical acceptance [11,12]. ER contributes to the development of the most frequent five cancers in females [13]. Thus, around a third of breast tumors are ER-positive and are characterized by slower growth, greater differentiation, a longer symptom-free survival interval and a higher sensitivity to endocrine therapy with antiestrogen drugs, such as tamoxifen [14]. The presence of ER and PR in the same tumor increases the likelihood of a response to hormonal treatment [15].

The c-erbB-2 gene encodes a 185 kDa transmembrane glycoprotein with intrinsic tyrosine kinase activity related to enhanced cell growth. This proto-oncogene is overexpressed in approximately 30% of breast cancer patients, indicating a poor prognosis, and is associated with drug resistance [16]. c-erbB-2 positivity has been associated with a significantly greater risk of endocrine therapy failure [15].

Antigen Ki-67 is a non-histone nuclear protein found in all phases of the cell cycle except for G0. High Ki-67 indexes are associated with low histological malignancy and with lymph node metastases [17]. Besides its usefulness as a cell proliferation marker, it has proven to be of prognostic value [18] in inverse correlation with ER receptors [19,20].

The protein encoded by the p53 gene functions as a master regulator of cell division and apoptosis programs [5]. The loss of its normal function is the most common genetic alteration in all types of cancer, and p53 is a central protein in tumorigenesis due to its cell-cycle and apoptosis regulating properties. It acts as nuclear transcription factor, binding to DNA in order to regulate the transcription of certain genes, and it monitors the DNA integrity, preventing the division of genetically damaged cells [21].

Breast cancer mortality has been substantially reduced over the past 15 years thanks to early detection strategies and advances in classical breast cancer therapy (surgery, chemotherapy, radiotherapy and hormone therapy). However, new forms of therapy are urgently needed to improve the long-term survival of patients with a poor prognosis, including gene therapy. One of the most promising recently developments has been the introduction of suicide genes that do not require the use of a prodrug to be effective in tumor cells. These genes, which encode cytotoxic products, include fusogenic membrane proteins such as vesicular stomatitis virus (VSV) glycoprotein and gibbon ape leukaemia virus (GALV) envelope protein [22,23]. When these proteins are expressed in a target cell, they cause membrane fusions with neighboring cells, leading to the formation of large multinuclear syncytia in which cells finally die by apoptosis or necrosis [24]. Our group previously reported that transfection of the *gef* gene from *E. coli*, identified as a member of a gene family that encodes homologous cell killing function [25], may be a new candidate for cancer gene therapy. Using the MCF-7 breast cancer cell line, we developed a new MCF-7 cell line transfected with a mammalian expression vector containing the *gef* gene (MCF-7TG) under control of the mouse mammary tumor virus (MMTV) promoter. Induction of gef expression leads to a marked decrease in cell growth rate, cell cycle arrest, membrane blebbing, craters on cell surface and cell death by apoptosis [26,27]. However, although the *gef* gene has shown considerable cytotoxic effects in breast cancer cells, the action mechanism has not been elucidated.

The present study demonstrates that *gef* gene not only reduces cell proliferation in transfected MCF-7TG cell line but also induces alteration of the most widely utilized breast cancer prognostic markers. Thus, *gef* gene induces an increase in ER and PR expressions and a decrease in Ki-67 and c-erbB-2 gene expressions, indicating a better prognosis and response to treatment and a longer disease-free interval and survival. It also increased p53 expression, suggesting that gef-induced apoptosis is regulated by a p53-mediated signaling pathway. These findings support the hypothesis that the *gef* gene offers a new approach to gene therapy in breast cancer.

## 2. Results and Discussion

Suicide gene therapy has been assayed in pre-clinical studies with various enzyme/prodrug systems, notably herpes simplex thymidine kinase gene (HSV-tk) [28,29]. The introduction of suicide genes that do not require the use of a prodrug to be effective in tumor cells, like the *gef* gene, represents a promising new development in this field [24,30,31]. The present study was designed to determine the mechanisms underlying the considerable cytotoxic effects of the *gef* gene in breast cancer cells, exploring the apoptosis pathway and the ability of the *gef* gene to affect the degree of maturation in breast cancer cells by studying its effects on a panel of specific breast tumor markers. Biochemical tumor markers are used to establish the diagnosis, prognosis and stage of the neoplasm, to detect the presence of hidden metastases and recurrences and to monitor the response to treatment. In this study, the most widely used prognostic markers for breast cancer were selected for inclusion in a prognostic panel capable of predicting the clinical behavior of the tumor, including its response to an individualized therapeutic regimen. The panel comprised ER, PR, erbB-2, antigen ki-67 and p53 protein [9].

### 2.1. Immunocytochemical Analysis

The immunocytochemical study demonstrated significant modifications in the antigenic expression pattern of cells transfected with *gef* gene compared with that of control cells. Percentages of stained cells and intensities of staining, obtained by microscopic study of control and *gef* gene-induced cell samples, are listed in Table 1.

**Figure 1:**
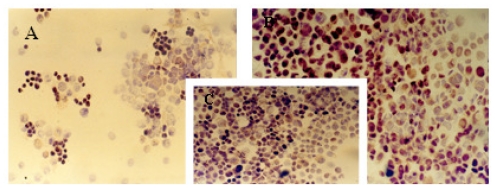
Immunocytochemical study with peroxidase staining of MCF-7 and MCF-7TG cells. Analysis of estrogen receptor (ER) expression in: A, parental MCF-7cells; B and C, MCF-7TG cells induced with dexamethasone (1 mM) for 2 and 6 days, respectively. (A, C, X 200 magnification; B X 400 magnification).

**Figure 2:**
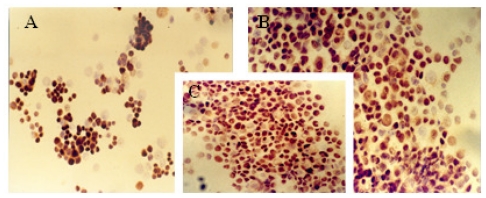
Immunocytochemical study with peroxidase staining of MCF-7 and MCF-7TG cells. Analysis of progesterone receptor (PR) expression in: (**A**) parental MCF-7cells; (**B**) and (**C**) MCF-7TG cells induced with dexamethasone (1 mM) for 2 and 6 days, respectively. (A, C, X 200 magnification; B X 400 magnification).

**Figure 3:**
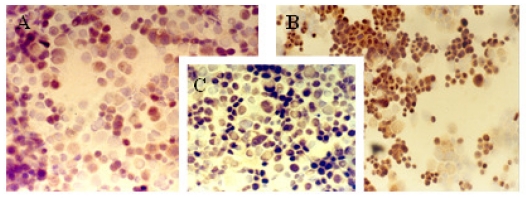
Immunocytochemical study with peroxidase staining of MCF-7 and MCF-7TG cells. Analysis of p53 antigen expression in: A, parental MCF-7cells; B and C, MCF-7TG cells induced with dexamethasone (1 mM) for 2 and 6 days, respectively, (A and C, X 400 magnification; B, X 200 magnification).

**Figure 4:**
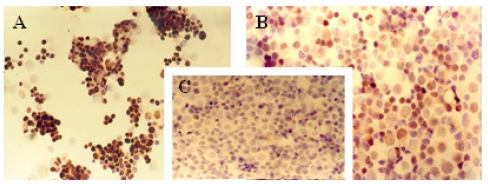
Immunocytochemical study with peroxidase staining of MCF-7 and MCF-7TG cells. Analysis of Ki-67 antigen expression in: A, parental MCF-7cells; B and C, MCF-7TG cells induced with dexamethasone (1 mM) for 2 and 6 days, respectively. (A, C, X 200 magnification; B X 400 magnification).

**Figure 5:**
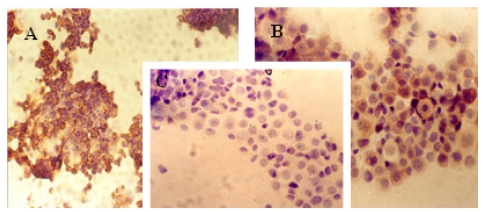
Immunocytochemical study with peroxidase staining of MCF-7 and MCF-7TG cells. Analysis of *c-erbB-2* oncogene expression in: A, parental MCF-7cells; B and C, MCF-7TG cells induced with dexamethasone (1 mM) for 2 and 6 days, respectively. (A, X 200 magnification; B and C, X 400 magnification)

Results obtained showed a slight increase in the expression of ER (Figure 1) and PR (Figure 2) hormonal receptors and a large increase in p53 (Figure 3) expression in the MCF-7TG cell line compared with the parental cells. However, Ki-67 antigens (Figure 4) and c-erbB-2 oncogenes (Figure 5) showed a significant reduction in the MCF-7TG cell line with respect to controls.

### 2.2. Indirect Immunofluorescence Analysis

**Figure 6:**
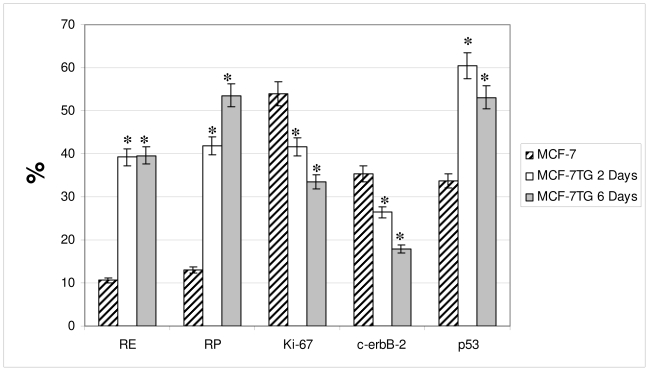
FACScan analysis of modifications in the expression of proteins in MCF-7TG cells treated with dexamethasone (1 mM) for 2 and 6 days. The asterisk means statistically significant.

Flow cytometry results confirm those obtained by the immunocytochemical study (Figure 6) and were as follows: *Estrogen receptor (ER)*: ER overexpression was observed in dexamethasone-treated MCF-7TG cells versus parental cells, with expression in 39.22% and 39.55% of MCF-7TG cells treated for 2 and 6 days, respectively, compared with 10.64% of parental cells.

#### Progesterone Receptor (PR)

PR overexpression was observed in MCF-7TG cells versus parental cells, with expression in 41.94% and 53.6% of MCF-7TG cells treated for 2 and 6 days, respectively, compared with 13.09% of parental cells.

#### Ki-67 Antigen

A significant reduction in Ki-67 expression was observed in MCF-7TG cells, with expression in 41.56% and 33.53% of MCF-7TG cells treated for 2 and 6 days, respectively, compared with 53.96% of parental cells.

#### p53 Protein

A significant increase in p53 expression was observed in MCF-7TG cells, with expression in 60.46% and 53.08% of MCF-7TG cells treated for 2 and 6 days, respectively, compared with 33.66% of parental cells.

#### c-erbB-2 Oncogene

A significant reduction in c-erbB-2 oncogene expression was observed in MCF-7TG cells, with expression in 26.42% and 17.97% of MCF-7TG cells treated for 2 and 6 days, respectively, compared with 35.37% of parental cells.

Numerous recent studies on the value of ER and PR as prognostic markers have supported their clinical relevance [9]. IHC procedures are used to determine their expressions in tumor tissue as indicators of the degree of proliferation, malignancy and invasion capacity of cancer cells [32]. ERs are intranuclear proteins, and their interaction with serum estradiol plays a key role in the regulation of breast epithelium proliferation and differentiation. ER is clinically relevant because its presence identifies tumors that are sensitive to hormone treatment with the corresponding antagonist. Thus, around half of metastatic breast cancers expressing ER and/or PR respond to endocrine therapy, and postoperative adjuvant endocrine therapy reduces the risk of recurrence by around 50% [15]. The role of PR is well documented, and it is widely accepted that ER+PR− patients have a more aggressive subtype of breast cancer with a poor prognosis, which may be related to a worse response to endocrine treatment [33,34]. In general, the presence of these hormonal receptors in breast carcinoma predicts a longer disease-free interval and survival [35]. Our demonstration that *gef* gene expression produces an increase in ER and PR levels implies a better response of these cells to hormonal treatment, since recurrence and survival rates are worse in ER− and PR-negative patients [34].

Various studies have demonstrated that 25–30% of all breast cancers overexpress *c-erbB-2* and that these tumors show higher tumor cell growth rates and more rapid progression to metastases versus tumors without overexpression of this oncogene [36,37]. *c-erbB-2* in breast carcinoma predicts time to recurrence, and its presence, in association with lower ER and PR levels and higher KI-67 levels, has been related to a lower responsiveness to endocrine therapy [15]. Its amplification is one of the most common genetic alterations associated with breast cancer, giving rise to resistance to chemotherapeutic drugs and to more aggressive clinicopathological behaviors [38]. Our finding of a progressive decrease in the expression of oncogene c-erbB-2 induced by *gef* in MCF-7TG cells indicates a better prognosis, since tumors overexpressing this oncogene have higher tumor cell growth and invasive potential versus those without this overexpression [36,37].

Expression of Ki-67 was also reduced in the MCF-7TG cells. This antigen is overexpressed in G_1_ and S phases but absent in resting cells and is used to estimate the intensity of proliferation. Ki-67 expression is considered of prognostic and diagnostic value in breast cancer as a cell proliferation marker [39,40]. When expressed in breast carcinoma, Ki-67 indicates tumor proliferation and therefore a poor prognosis. Hence, the reduced Ki-67 expression in MCF-7TG cells appears to support the antiproliferative effect of *gef* gene in breast cancer cells [26,27], although the relationship between expression of this antigen and metastatic capacity is controversial [41].

Our immunocytochemical study showed that the p53 tumor suppressor gene was significantly up-regulated by induction of *gef* gene. The p53 gene intervenes in the cell cycle, negatively regulating tumor growth, and its overexpression is known to be induced by DNA damage, producing cell arrest in G_1_ phase for repair or entry into apoptosis [5]. Numerous studies have addressed the prognostic or predictive significance of p53 in breast carcinomas but controversial results have been published [42–44]. Most clinicopathological studies have used IHC techniques to estimate p53 accumulation, some affirming [45,46] and others refuting [47,48] the predictive value of p53, which may be of critical importance for breast carcinoma treatment, especially chemotherapy. Studies on the prognostic significance of p53 mutation found it to be a significant independent negative factor for disease-free and/or overall survival [7,49]. The effects of p53 on cell growth, arrest and apoptosis and on the maintenance of genomic integrity are highly complex. The effects of distinct p53 mutations are likely to differ in their influence on tumor development, growth and prognosis [50]. Mutation of the gene, with IHC-detectable protein accumulation in cancer cells, is associated with a poor prognosis in breast cancer. It is correlated with an absence of hormone receptors, presence of receptor for Epidermal Growth Factor (EGF) and a more aggressive tumor [51]. However, the p53 overexpression detected by IHC is not always related to p53 gene mutations [52]. It also detects accumulation of wild-type p53 [53], whose accumulation is frequently observed in normal tissues and benign neoplasms in response to a specific cell cycle phase or to spontaneous genetic errors or microenvironmental stresses on individual cells. The p53-encoded protein participates in the apoptotic pathway by inducing cell cycle arrest and initiating apoptosis, which were previously reported as anti-cancer effects of *gef* gene expression on the MCF-7 cell line [26,27]. However, the mechanism of action has not been elucidated. The present finding of a significant upregulation of p53 gene suggests that gef-induced apoptosis is regulated by a p53-mediated signaling pathway. Our results are similar to those obtained by Seo *et al*., [54] after treatment of the same breast cancer line with PEGylated conjugated linoleic acid.

## 3. Experimental Section

### 3.1. Cell Lines and Culture Conditions

The human breast cancer MCF-7 cell line was kindly provided by Mr. N. Olea of the Sánchez Mora Tumour Biology Institute, San Cecilio University Hospital of Granada. MCF-7 cells were grown at 37 °C in an atmosphere containing 5% CO_2_, with Dulbecco’s modified Eagle Medium (DMEM) (Gibco, Grand Island, NY, USA) supplemented with 10% heat-inactivated foetal bovine serum (FBS) (Gibco), 2% L-glutamine, 2.7% sodium bicarbonate, 1% Hepes buffer, 40 mg/L gentamicin and 500 mg/L ampicillin.

Human breast cancer MCF-7TG cells were obtained by transfection of MCF-7 cell line with *gef* gene as we describe in previous work [26].

### 3.2. Immunocytochemical Analysis

Adherent parental MCF-7 cells and MCF-7TG cells treated with dexamethasone for 48 h and 96 h were harvested with PBS-EDTA (0.02%), washed twice with PBS at 1500 rpm for 5 min at 4 °C, and resuspended in 0.5 mL of PBS. These cells were fixed to slides by high-speed centrifugation, and the slides were subsequently dried in room air, fixed for 60 seconds with 70% methanol, and frozen to −20 °C. The immunocytochemical analysis was done using ChemMate Kit (Dako) according to manufacturer’s instructions. The slides were thawed, washed with PBS, incubated in peroxidase-inhibition solution for 5 min. They were then washed in PBS three times, and after that incubated with primary antibody (see below) for 1 h in a dark humid chamber. Next, they were washed with PBS three times, incubated with secondary antibody (AB2) for 30 min in a dark humid chamber, and washed again three times with PBS. The slides were incubated with conjugated antibody for 25 min, washed twice, first with PBS and then with water, and developed with diaminobenzidine (DAB) until appearance under microscope of the desired staining. Once stained, slides were washed three times with distilled water and then in rising concentrations of ethanol (from water to 96% ethanol). Readings were done under optical microscope and interpreted by the same observer. The antibodies used are listed in Table 2.

### 3.3. Indirect Immunofluorescence Analysis

The *gef* gene was induced in MCF-7TG cells with dexamethasone for 48 or 96 h. Parental MCF-7 cells with or without dexamethasone incubation were used as controls. Cells were harvested in PBS-EDTA (0.02%), washed twice in cold PBS, counted and pelleted by centrifugation at 1500 rpm for 5 min at 4 °C, and 6 × 10^5^ cells were incubated with 10% (w/v) foetal calf serum in PBS for 15 min to block non-specific antibody binding. Then, pellets were resuspended in 20 μL of the monoclonal antibody. The resulting solution was shaken and incubated for 30 min in the dark at 4 °C. Then, after being washed once with cold PBS and centrifuged at 1500 rpm for 5 min, the supernatant was removed and 15 μL of fluorescein-conjugated antiimmunoglobulin (Sigma, St. Louis, MO) diluted to 1:5 or 1:10 was added to the pellets. They were resuspended and incubated for 30 min at 4 °C and, after two washes with cold PBS, the supernatant was decanted and cells were fixed with 70% ethanol. Cells were then processed using a FACScan flow cytometer (Becton Dickinson, San Jose, USA). The antibodies used are listed in Table 2.

The final expression of the specific fluorescence for each antibody was corrected for autofluorescence and non-specific fluorescence. All experiments were performed in quadruplicate and yielded similar results.

### 3.4. Statistical Analysis

SPSS version 7.5 [55] was used for the statistical analysis. The results were compared by means of the Student’s *t* test. All data are expressed as means ± SD. Differences were considered statistically significant at a *P* value of < 0.05.

## 4. Conclusions

Induction of *gef* gene expression in breast cancer cells produced changes in specific tumor markers that indicated a better prognosis and response to treatment, with an increase in the disease-free interval and survival rate. Thus, c-erbB-2 and Ki-67 expressions were lower in *gef* gene-transfected MCF-7 breast cancer cells, predicting a reduced proliferation rate and metastasizing capacity, and ER and PR expressions were higher, predicting a better response to hormonal treatment. Finally, the upregulation of p53 in MCF-7TG cells suggests that gef-induced apoptosis is regulated by a p53-mediated signaling pathway. Overall, the anti-cancer effects of *gef* gene on MCF-7 breast cancer cells may be of therapeutic value. We are currently working on experiments to enhance gef gene activity by specific enhancer/promoter genes (such as tyrosinase) to induce tissue-specific expression. The possibility of using selective transcriptional control sequences with *gef* therefore offers the gene therapist a tool of significant potential in which the use of a prodrug is not necessary.

## Figures and Tables

**Table 1:** Percentages of stained cells and intensities of staining, obtained by microscopic study of control and gef gene-induced cells treated with dexamethasone (1 mM) for 2 or 6 days.

Cell line	Protein markers									

	ER		PR		c-erbB-2		Ki-67		p53	
	
	A	B	A	B	A	B	A	B	A	B
**MCF-7**	13%	++	20%	++	62%	+++	72%	+++	33%	++
**MCF-7TG dex 2 days**	50%	++	57%	++	27%	+	18%	++	59%	+++
**MCF-7TG dex 6 days**	55%	++	61%	+	17%	+	8%	+/−	55%	+++

A: Percentage of stained cells; B: Degree of intensity; −: negative; +: weak staining; ++: moderate staining; +++: intense staining.

**Table 2:** Antibodies used in the Immunocytochemical and Indirect Immunofluorescence analysis.

Antibody	Source	Dilution	Staining pattern in cancer cells
**ER**	Dako (Barcelona, Spain)	1:200	Nuclear staining
**PR**	Dako (Barcelona, Spain)	1:250	Nuclear staining
**c-erbB-2**	Dako (Barcelona, Spain)	1:250	Membrane staining
**Ki-67**	Dako (Barcelona, Spain)	1:100	Nuclear staining
**p53**	Dako (Barcelona, Spain)	1:100	Nuclear and Cytoplasm staining
